# Dehydroepiandrosterone sulphate: diabolical hormone or epiphenomenon in aneurysmal subarachnoid hemorrhage?

**DOI:** 10.1186/s13054-015-1069-4

**Published:** 2015-10-06

**Authors:** Santosh B. Murthy, Neeraj S. Naval

**Affiliations:** Division of Neurosciences Critical Care, Johns Hopkins University School of Medicine, 600 N Wolfe St, Phipps 455, Baltimore, MD 21287 USA

## Abstract

Inflammation is purported to play an important role in the clinical course of subarachnoid hemorrhage. The current study by Höllig et al. entails using dehydroepiandrosterone sulfate, a hormone that inhibits key inflammatory pathways, as a predictor of functional outcome in these patients.

Attempts to incriminate inflammation as the major predictor of clinical outcomes following aneurysmal subarachnoid hemorrhage (SAH) have been met with disappointment. Moreover, clinical trials that have focused on ameliorating cerebral vasospasm, thought to be induced by an inflammatory cascade [1], using statins, endothelin antagonists, aspirin, magnesium, steroids, and nonglucocorticoid free radical scavengers have been largely unsuccessful [2–8].

In a recent article in *Critical Care*, Höllig et al. [9] attempt to add a new dimension to our understanding of inflammation in SAH; that is, a hormonal twist—enter dehydroepiandrosterone sulfate (DHEAS). The authors prospectively performed serial measurements of serum interleukin (IL)-6 and DHEAS in 53 SAH patients. Functional outcome was evaluated using the modified Rankin Score (mRS) at discharge and 6 months, dichotomized into favorable outcome (mRS 0–2) and poor outcome (mRS 3–6). The study was centered on the hypothesis that DHEAS has a neuroprotective role in SAH, primarily by inhibition of IL-6, one of the key cytokines incriminated often in neuroinflammatory pathways. A significant correlation between DHEAS and IL-6 levels and outcomes was noted by the authors.

We commend the authors on undertaking this complex, yet important study. A noteworthy observation is that mean levels of DHEAS appeared to plummet between days 4 and 7, before trending up again. Conversely, mean levels of IL-6 peaked in the corresponding time frame, particularly in patients with poor outcome. This timeline coincides with the onset of vasospasm [10], although this relationship was not explored in the current study. The ambitious follow-up period of 6 months is also conceptually one of the strengths, as compared with 3 months in most studies [11]. While this extended time period allows for better neurological recovery and hence more accurately predicts functional outcome, the study size significantly suffered because of loss to poor follow-up.

Before conducting larger scale validation studies in future, a few important questions need review. First, do levels of central nervous system inflammatory makers correlate with serum levels? In other words, are we sampling the right source to study inflammation in these patients? Even central inflammation, for instance, has three possible substrates, namely the meninges/cerebrospinal fluid, brain parenchyma, and cerebral arteries, with varying severity of inflammation [12]. This has an important bearing on the interpretation of the results since the majority of the studies, including that by Höllig et al., focus on using peripheral inflammation as a surrogate for central nervous system inflammation.

Second, is the inflammation truly a predictor of outcome or merely a marker for neuronal injury? This question has remained an enigma for decades. While the mechanistic role of inflammation in predicting complications or outcomes in SAH has yet to be elucidated, Höllig et al. attempt to add another variable to the equation by bringing in DHEAS. The causal role of DHEAS in influencing SAH outcomes trespasses into the realm of inflammation, at which point its role as an outcome predictor or merely an epiphenomenon becomes murky.

In conclusion, despite an obvious cause–effect relationship between DHEAS and outcomes, the observed link between the aforementioned levels and outcomes is an important one. DHEAS levels may not only help in prognostication of SAH outcomes, but could potentially help in vasospasm surveillance if a relationship were to exist. Further, if DHEAS truly is a driver or modifier of inflammation in SAH, this could be an exciting new therapeutic target for future research.

## Abbreviations

DHEAS: Dehydroepiandrosterone sulfate
IL: Interleukin
mRS: Modified Rankin Score
SAH: Subarachnoid hemorrhage
